# Tracking Population-Level Anxiety Using Search Engine Data: Ecological Study

**DOI:** 10.2196/44055

**Published:** 2023-03-22

**Authors:** Barnabas James Gilbert, Chunling Lu, Elad Yom-Tov

**Affiliations:** 1 Department of Brain Sciences Imperial College London London United Kingdom; 2 Department of Global Health & Social Medicine Harvard Medical School Boston, MA United States; 3 Microsoft Research Herzliya Israel

**Keywords:** anxiety disorders, anxiety themes, Bing search, country-level, epidemiology, Google trends, internet search data, mental disorder, search engine, socioeconomic

## Abstract

**Background:**

Anxiety disorders are the most prevalent mental disorders globally, with a substantial impact on quality of life. The prevalence of anxiety disorders has increased substantially following the COVID-19 pandemic, and it is likely to be further affected by a global economic recession. Understanding anxiety themes and how they change over time and across countries is crucial for preventive and treatment strategies.

**Objective:**

The aim of this study was to track the trends in anxiety themes between 2004 and 2020 in the 50 most populous countries with high volumes of internet search data. This study extends previous research by using a novel search-based methodology and including a longer time span and more countries at different income levels.

**Methods:**

We used a crowdsourced questionnaire, alongside Bing search query data and Google Trends search volume data, to identify themes associated with anxiety disorders across 50 countries from 2004 to 2020. We analyzed themes and their mutual interactions and investigated the associations between countries’ socioeconomic attributes and anxiety themes using time-series linear models. This study was approved by the Microsoft Research Institutional Review Board.

**Results:**

Query volume for anxiety themes was highly stable in countries from 2004 to 2019 (Spearman r=0.89) and moderately correlated with geography (r=0.49 in 2019). Anxiety themes were predominantly long-term and personal, with “having kids,” “pregnancy,” and “job” the most voluminous themes in most countries and years. In 2020, “COVID-19” became a dominant theme in 27 countries. Countries with a constant volume of anxiety themes over time had lower fragile state indexes (*P*=.007) and higher individualism (*P*=.003). An increase in the volume of the most searched anxiety themes was associated with a reduction in the volume of the remaining themes in 13 countries and an increase in 17 countries, and these 30 countries had a lower prevalence of mental disorders (*P*<.001) than the countries where no correlations were found.

**Conclusions:**

Internet search data could be a potential source for predicting the country-level prevalence of anxiety disorders, especially in understudied populations or when an in-person survey is not viable.

## Introduction

Anxiety disorders are the most prevalent mental disorders globally [1]. The 2019 Global Burden of Disease Study estimated a consistent increase in the prevalence, incidence, and disability-adjusted life years burden of anxiety disorders at global, regional, and country levels between 1990 and 2019 [1]. The total number of people diagnosed with anxiety disorders increased from 195 million in 1990 to 301 million in 2019 [1]. The COVID-19 pandemic has added to the global burden of anxiety disorders with global prevalence increasing by over 25% in 2020 to 378 million [2]. The prevalence of anxiety disorders has increased the most in the countries most affected by the COVID-19 pandemic, as determined by daily infection rates and reductions in human mobility [2]. The situation could worsen further in the case of a global economic recession [2].

Anxiety disorders are associated with chronic physical disorders (eg, chronic ischemic heart disease) and frequently precede the onset of other mental disorders and suicide [3]. Besides the immense negative impact on individual function and quality of life, they impose a significant economic burden on families and societies [1]. A range of pharmacological, psychological, and social interventions have been proven to be cost-effective in diagnosing and treating anxiety disorders [1]. Regrettably, due to resource shortages allocated to mental health care, many patients have not had access to appropriate diagnosis or treatment [4]. Information on what contributes to symptoms of anxiety disorders and their temporal and geographic trends is necessary to efficiently spend limited funds on preventive and treatment interventions, and to enable interventions to be customized and targeted to the areas of greatest need.

This study aimed to track the trends in anxiety themes between 2004 and 2020 in the 50 most populous countries with high volumes of internet search data. Between 2004 and 2020, internet use and search activity increased significantly in all countries [5], marking the “democratization” of the internet. Internet search data are known to reflect the behaviors, activities, and concerns of people in the physical world [6]. Tracking patterns in internet search data have become an effective and widely used research tool for understanding population-level behavior, with the potential to inform and facilitate preventive and treatment strategies for a wide range of health disorders. For example, such data have been used to track both physical and mental disorders [7,8] and have previously been published in this journal in relation to anxiety and other disorders [9,10]. The authors have recently applied similar research methods to other mental health studies [11]. To date, most studies using search data to track anxiety themes or determinants have focused on limited time spans (eg, the COVID-19 pandemic) [12] and geographic diversity (eg, France) [13].

This study extends previous research by using a novel search-based methodology and including a longer time span and more countries at different income levels. The study identified themes associated with anxiety in different countries through a semistructured crowdsourced questionnaire, mapped them to the terms used in internet search queries, and generated the volume of these terms across time and geography.

## Methods

### Identifying Anxiety Themes Through a Crowdsourcing Survey

Search engine queries are often short and without context, which makes it difficult to know if all queries which use a possibly relevant term are related to anxiety. To address this issue, our study adopted a novel method, as described below. We began our investigation by conducting a crowdsourced survey of anxiety themes. The survey was constructed in a semistructured format, with 4 questions about what makes a respondent or his/her friends feel anxious in the short and long term (see Multimedia Appendix 1). The survey was conducted on the Amazon Mechanical Turk platform. Due to time and financial constraints, we gathered data from the first 200 people providing answers to the survey questions. The answers were manually grouped by the authors into anxiety themes in the short or long term. Each anxiety theme was further classified by the authors into the following three domains: personal, familial, or societal. Information generated from the crowdsourced survey could provide real-world context on factors that respondents associate with anxiety, phrased in a manner they find natural and intuitive. Annotator agreement, as measured by Cohen kappa, was 0.62 for the anxiety theme and 0.77 for domain, meaning that interrater reliability for manual grouping by the authors was substantial in both cases.

### Mapping Anxiety Themes Obtained From the Survey to the Terms Used in Bing Data

We investigated which anxiety themes obtained from the survey were likely to be related to the terms queried in the Bing search engine. First, we translated (using the Google Translate service) the themes identified through crowdsourcing into the 7 most common languages across the world, aside from English [14]. These include Mandarin Chinese, Spanish, Hindi, Bengali, Portuguese, Russian, and Japanese. We then used all queries submitted to the Bing search engine during 2020 to assess the correlation between the themes identified through crowdsourcing and terms used in the Bing search that might be related to anxiety. Specifically, we counted the number of times each appeared (in any of the 8 languages) without context (eg, “childbirth”) and in context together with keyword(s) related to anxiety (eg, “I am anxious about childbirth”). For the latter, we included the keyword(s) “anxious about,” “worried about,” “nervous,” “dread,” “panic,” “bothered by,” “concerned,” “distressed,” “frightened,” and “tormented.”

We focused on the 50 terms which had the highest correlation between the number of times they were mentioned in context and the number of times they were mentioned without context. The need to assess search volume without context is because Google Trends data do not contain the context of queries. We validated the identification strategy by comparing the fraction of mentions of terms without context (eg, “childbirth”) to that with anxiety-specific contexts (eg, “I’m anxious about childbirth”). Further details can be found in Figure A1 in Multimedia Appendix 1.

### Extracting Trends in Anxiety Themes Through Google Trends

Google Trends is a website where users can type a search query or a search topic and be provided with the volume of searches for the query or topic (ie, the number of queries made) in different times and geographies. We refer to this value as the search volume. Search topics, known as Google Trends terms (GTTs), are topical groupings of multiple queries across languages. These groupings overcome the need to collect Google Trends data for individual queries and to translate these queries to all relevant languages. Note that Google Trends provides data normalized to the range of 0-100. As we are interested in the volume of the queries, we thus used the Google Trends Anchorbanks interface, which provides unnormalized Google Trends data [15].

The authors manually mapped themes that were associated with anxiety, including when mentioned without context, to their corresponding GTTs. Only 37 of the mapped terms had corresponding GTTs. We therefore used these 37 terms in the analysis and excluded the remaining 13 (see Table A1 in Multimedia Appendix 1). From hereon, “themes” will be used to refer to these mapped search terms with corresponding GTTs, given this is what they are a proxy for.

The search volume in the 50 most populous countries (see Table A2 in Multimedia Appendix 1) [16] was extracted at a monthly resolution from January 2004 to December 2020. Among the 50 countries, 7 were low-income countries, 16 were lower-middle income, 12 were upper-middle income, and 15 were high-income countries, according to the World Bank income classification in 2016 [17]. We focused on these most populous countries so that sufficient search data would be available.

### Analysis

We generated the annual volume for each of the 37 anxiety themes in each country using Google Trends data from 2004 to 2020. We grouped themes by 3 domains (personal, family, and country) and time frame (short-term and long-term) to show which types of themes had the highest query volume over time and across countries. The information is important for policy makers and other stakeholders to identify related preventive and treatment strategies in their countries.

We used Spearman correlation to investigate if the query volume for anxiety themes is correlated over time or across the 50 countries. We also measured the similarity between countries by calculating the correlation of volumes of anxiety themes between the countries.

To estimate whether the total volume of anxiety themes varied over time in each country, we calculated the total monthly volume of anxiety themes and then clustered this time series using k-means clustering with 3 clusters and correlation similarity. The number of clusters was set to the smallest number that did not create clusters with fewer than 5 countries. The centroids of these clusters show how the total volume of anxiety themes changed over time in each country.

We investigated into the possibility of compensatory and noncompensatory effects that described the association between the changes in volume of the most searched themes and the changes in volume of less frequently searched themes. Compensatory effect was defined as the situation where a rise in the volume of the most searched anxiety themes was associated with a decrease in the volume of less frequently used search themes. A noncompensatory effect was defined as the situation where the rise in the volume of the most searched anxiety themes was associated with an increase in the volume of less frequently used searched themes. For each country, we first selected 3 most searched anxiety themes by year and summed up their monthly volumes. We selected the 3 most searched terms because terms could be correlated (eg, childbirth and maternity), and it is necessary to compare several of the most voluminous themes. We then generated the sum of monthly volume by year for the remaining themes. To detect the presence of the 2 types of effects, we applied a time series cross-country OLS model with the sum of monthly volume for the less frequently searched themes by country and year (denoted by *Y*_it_) as the dependent variable:

Y_imt_ = β_0_ + β_1_X_imt_ + β_2_C_i_ + β_3_Year*_t_* + β_4_X_imt_*C_i_ + ε_imt_

Here, *X*_imt_ denotes the sum of the monthly volume of the 3 most searched anxiety themes in country *i* at month *m* and year *t*, *C*_i_ is a country indicator variable, Year*_t_* is an indicator for year *t*, * denotes the interaction, and *ε*_imt_ indicates random error. If there is a compensatory effect in a country, we expect a negative coefficient of the interaction variable in that country: the country’s monthly volume of the 3 most searched anxiety themes was inversely associated with its monthly volume of the remaining themes. If there is a noncompensatory effect in a country, we expect a positive coefficient of the interaction variable (*β*_4_) in that country.

We also investigated how anxiety themes were associated with countries’ attributes such as economic development, demography, politics, religion, and culture. To process the analysis, we gathered the country-level data, as listed in Table 1. Country-level attributes are defined in Multimedia Appendix 1. For each country, we examined the correlation between the volume of each of the anxiety themes and each of the country-level attributes. We also investigated the association between the type of effects (compensatory vs noncompensatory) and countries’ attributes using correlation tests.

**Table 1:** Country-level attributes and data sources.

Country-level attributes	Reference
Gross domestic product	[18]
Life expectancy	[19]
Median age	[19]
Percent of urban population	[20]
Sex ratio	[20]
Sex and age standardized prevalence of mental disorders	[21]
Global Freedom scores	[22]
Religiosity	[23]
Fragile States Index	[24]
The 6 cultural dimensions of countries	[25]

### Ethics Approval

This study was approved by the Microsoft Research Institutional Review Board (IRB: #10219). Written informed consent (through a web form) was obtained for the crowdsourcing questionnaire. Other data were anonymous and aggregated.

## Results

### Overview

Among 200 responses for a crowdsourced questionnaire to identify anxiety themes, a total of 184 valid responses were collected, coming from respondents in 15 countries, with the majority from the United States and Brazil. Overall, 95 anxiety themes were identified by the survey.

### Total Volume of Anxiety Themes Over Time

Table A2 in Multimedia Appendix 1 presents the anxiety themes with the highest volume by country and year. Between 2004 and 2019, themes with the highest volume that were most frequently presented among the 50 countries include worrying about “having kids,” “pregnancy,” and “job” regardless of a country’s income level and region. For example, worrying about “job” was found with the highest volume in countries from the low-income group (eg, Afghanistan, Nepal, Tanzania, and Uganda), lower-middle income group (eg, Bangladesh, Ghana, India, Kenya, Myanmar, Nepal, and Nigeria), upper-middle income group (eg, Malaysia and South Africa), and high-income group (eg, the United Kingdom and the United States). In 2020, worrying about “COVID-19” became the theme with the highest query volume in more than half of the countries.

Clustering of the total volume of anxiety themes showed that it was reduced between 2004 and 2014, after which it remained approximately constant for 22 countries. The total volume remained approximately constant over the entire period in 9 countries. The volume in the remaining 19 countries increased, especially after 2014.

Among 50 countries, only those with a lower fragile state index (*P*=.007) or a higher individualism index (*P*=.003) were significantly more likely to be in the group with constant total volume of anxiety themes over time.

### Correlation of Volumes of Anxiety Themes Between Years and Across Countries

The volume of anxiety themes was highly correlated over time and, to a lesser extent, geography: Spearman correlation between the search volume for anxiety themes in 2004 and in 2019 was, averaged across all countries, 0.89. We did not include the data after 2019 in this part of the analysis to avoid the pandemic’s shock on the trends.

In 2019, the average correlation in query terms across the 50 countries was 0.49. Figure A2 in Multimedia Appendix 1 shows that some geographically proximate countries are more similar in the volume of query terms (eg, Germany and Italy).

We also examined the correlation between the volume of the most voluminous theme in 50 countries and countries’ anxiety incidence reported by the Global Burden of Disease Project. The correlation was statistically significance, although at a low level (Pearson *r*=0.28, *P*=.047).

### Volumes of Anxiety Themes by Longevity and Domain

**Figure 1:**
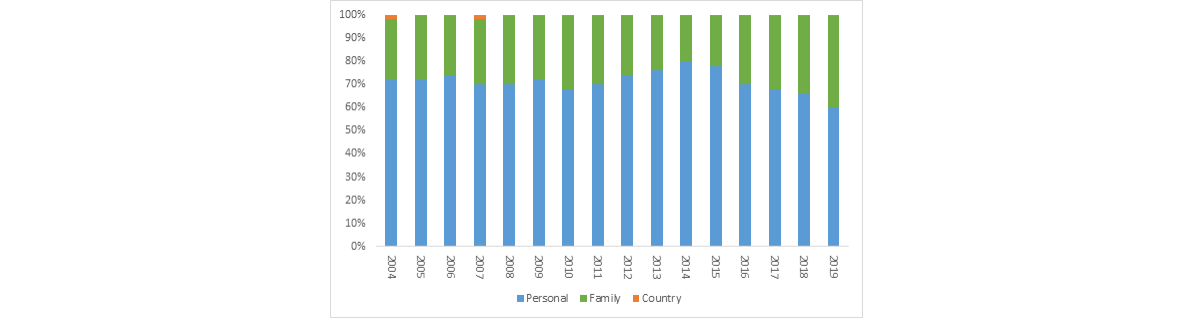
Anxiety themes by domain over time.

**Figure 2:**
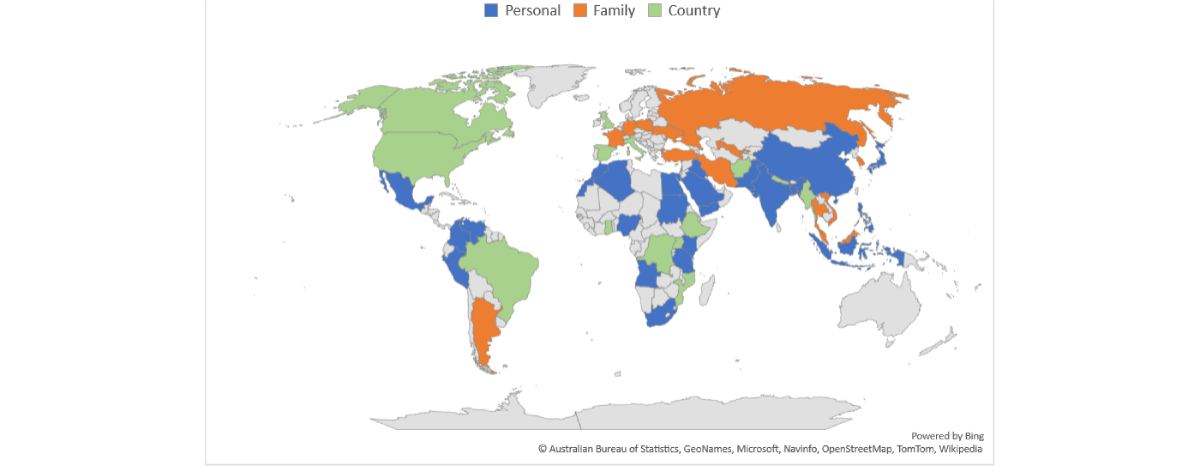
The most voluminous domain in 50 countries in 2020.

In almost all countries, the anxiety themes with the largest volume were long-term themes (eg, chronic illness) rather than short-term themes (eg, job interviews) in all years. Figure 1 shows the trends of the anxiety themes with the largest volume across the 3 domains between 2004 and 2019. Over time, anxiety themes with the highest volume were those related to personal matters. Figure 2 presents the domain with the highest volume in 50 countries in 2020 when the COVID-19 pandemic started. The share of anxiety themes related to country-level matters increased sharply, accounting for the largest volume of anxiety themes in 14 countries with different income levels (eg, Brazil, Canada, China, Ethiopia, India, the United Kingdom, and the United States).

### Association Between Country Attributes and Query Volume of Anxiety Themes

No country attributes were found to be significantly correlated with the query volume except for 3 cultural dimensions, which had weak correlations: power distance (*ρ*=0.14, *P*=.004), masculinity (*ρ*=–0.15, *P*=.002), and long-term orientation (*ρ*=–0.14, *P*<.001).

### Compensatory and Noncompensatory Effects in Each Country

**Figure 3:**
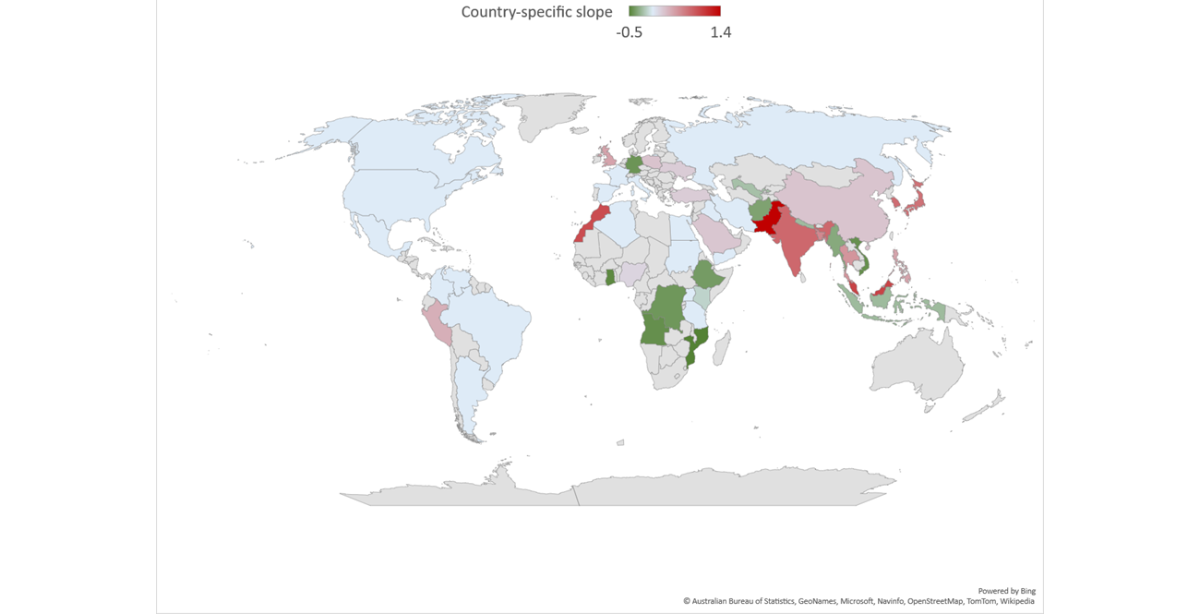
Association in search volume of less voluminous themes with that of search volume in the 3 most voluminous themes. Countries in green (13 countries) exhibit a compensatory effect, and countries in red exhibit a noncompensatory effect (17 countries). Blue denotes countries with nonstatistically significant slopes, and countries in gray are not included in this study.

Figure 3 shows the coefficient of country-specific interactions derived from the regression model. Thirteen countries exhibited a compensatory effect, where a rise in the volume of the 3 most voluminous themes was associated (to varying degrees) with a fall in the volume of the remaining themes; 17 countries exhibited a noncompensatory effect, where a rise in the volume of the 3 most voluminous themes was associated (to varying degrees) with a rise in the volume of the remaining themes; and the remaining 20 countries exhibited no effects.

Among country-level attributes, only the prevalence of mental disorders was associated with the partition shown in Figure 3 (Kruskal-Wallis, *P*<.001, significant after Bonferroni correction). Multiple comparison reveals that the countries without significant interaction effects had a significantly higher prevalence of mental disorders than the 2 other groups of countries.

## Discussion

Our findings suggest anxiety themes tend to be stable over time within and, to a lesser but significant extent, between countries. In almost all countries, themes most searched for were long-term factors, suggesting that anxiety might be more commonly driven by chronic environmental stressors and that people with an anxiety disorder are more likely to ruminate on long-term factors.

In cognitive behavioral theory, such relationships are understood in terms of the dynamic, cyclical interactions between emotional feelings of anxiety and their associated thoughts, somatic perceptions, and behaviors [26]. While correlation with anxiety disorder subtypes has not been examined in this study, evidence suggests that chronic, pervasive anxiety disorders such as generalized anxiety disorder are more common than anxiety disorders triggered acutely, such as panic disorder [27].

Our finding that the highest volume anxiety themes pertain to personal, rather than familial or country level, themes is novel in the literature. It is supported by survey findings that “finance, money, and debt” are the most common source of anxiety [28], although these economic factors can be argued to exist ambiguously across personal, familial, and country-level domains.

Anxiety themes regarding country-level matters became more prevalent during the COVID-19 pandemic. This contrasts with prior cross-sectional analyses showing that higher personal COVID-19 exposure was positively correlated with anxiety symptoms, but country-level COVID-19 factors were not. Other studies have found that factors negatively correlated with mental health during COVID-19 were predominantly personal: self-isolation, false beliefs, and a weak sense of coherence [29]. This inconsistency in findings requires further investigation. Future work will analyze the themes of anxiety throughout the pandemic and compare it to pre- and postpandemic themes.

Prior studies have shown that social anxiety among students is positively correlated with individualism [30], although this depends on whether the personality traits of the individual reflect or conflict with the cultural values of the society they are currently living in [31]. No mental disorders have been reliably shown to increase in prevalence with increasing socioeconomic status.

Volume of anxiety-related themes was not found to be associated with the other country attribute variables in this study. Burkova and colleagues [31], by contrast, identified cultural looseness/tightness, alongside individualism/collectivism and power distance, as cultural dimensions which may protect against the emergence of anxiety symptoms during the COVID-19 pandemic. The lack of association with sex ratio may warrant further investigation, given that the higher prevalence of anxiety disorders in women has been well established [32]. Similarly, on median age, Global Burden of Diseases studies on anxiety have shown that younger age groups tend to be affected more frequently than older age groups [1,2].

A prior study in Europe and the United States found that freedom index was negatively correlated with anxiety and stress symptoms, which the investigators ascribed to greater autonomy and opportunities for happiness [33]. Lack of religiosity has previously been associated with increased anxiety symptoms in older adults, though not in the young or middle-aged people. Generalized anxiety disorder has been shown to increase by more than 20% in urban populations [34]. Life expectancy has previously been shown to decrease, and mortality to increase, in people with anxiety disorders [35].

Certain geographically proximate countries were found to be more similar in the volume of query terms over time (see Figure A2 in Multimedia Appendix 1), although the themes could not be identified in our present analyses. Prior investigators have hypothesized that differences in the prevalence of anxiety disorders between countries and time periods increase the weighting of environmental influences in their etiology, whereas when prevalence rates are stable, despite different socioeconomic and cultural environments, the weighting of genetic and neurobiological influence increases [36]. We hypothesize that the variation identified in search volumes for different anxiety themes between countries is due to different environmental stressors (most likely personal and long-term) present in distinct socioeconomic and cultural settings.

Our analyses reveal that anxiety themes can interact in the following three ways: an increase in a dominant anxiety theme is either associated with an increase in other themes, a decrease therein, or is unrelated. The finding that countries without interaction effects had higher prevalence of mental disorders than the other 2 groups of countries suggests that understanding the interactions between the volumes of anxiety themes could help predict a country’s prevalence of mental disorders and inform the development of predictive models of anxiety at the population level.

Our study has several limitations, including selection bias, as Mechanical Turk participants who opted-in to the initial survey were not prescreened for historic or active diagnoses of anxiety or other mental disorders; language bias, due to exclusion of noncommon languages, not validating automated translations with native speakers, and not accounting for multiple language use within countries; use of a nonexhaustive list of keywords related to anxiety; the inability to precisely match all the selected terms from Bing search data in Google Trends; the inability to split our analyses by sex and age; differences in search engine usage across countries and over time and how Google computes Google Trends scores; and limited internet access in low-income countries. Additional work is required to validate our findings using both traditional and novel research methodologies. Further work could also make use of related searches and topics in Google Trends.

Globally, the proportion of the population using the internet, including for search engine use, increased dramatically between 2004 and 2020 [36]. Over time, populations with different demographic and socioeconomic backgrounds could have different levels of access to the internet and of using the internet. Evidence has shown that the proportion of internet use among elderly, low-income, or low-education populations was much lower than their counterparts. For example, in Ethiopia, internet usage increased with the level of education: only 5% of men with primary education reported ever using the internet, compared with 68% of men with more than secondary education; and 16% of women in the highest income quintile, compared to 0.1% in the lowest income quintile [37]. Internet use in the same population across years could also vary: among those aged 65 years and older in the United States, internet users increased from 14% in 2000 to 73% in 2017 [38]. Further work should examine how such changes in the demographic and socioeconomic characteristics of internet use could impact general patterns of anxiety themes. As research indicates that lower household income is associated with increased risk for mental disorders [39] and the lowest income groups within communities are up to 3 times more likely to suffer from anxiety and other common mental disorders than those with the highest incomes [40], it is very likely that low-income groups may be substantially underrepresented in the search data in this study.

For policy makers in a country, national-level data may only be of limited use in making resource allocation decisions. Disaggregating national data by individual demographic and socioeconomic characteristic (eg, gender, income level, and education) and by region with different levels of development will equip the policy makers with better evidence for understanding the disparities in the themes of anxiety and identify the most vulnerable populations for policy support. Future work should focus on defining and developing an approach to collect and analyze disaggregated data in countries of interest and address issues such as availability and consistency of data between different population groups and regions.

Nevertheless, we believe that the use of internet data to study the drivers of mental health concerns across multiple countries and over long durations opens a new direction for studies of entire populations, especially in understudied populations or during pandemics (eg, COVID-19) when in-person survey is not viable. Our search-based methods enable real-world perception of anxiety associations to be identified at scale, which may be an important step in digital phenotyping, predictive modeling, and preventive interventions for anxiety.

## Appendix

Multimedia Appendix 1 Supplementary information.

## Abbreviations

GTT: Google Trends term

## Data Availability

The questionnaire and search engine data generated and analyzed during the current study is available from the corresponding author on reasonable request with Microsoft’s approval. Google Trends data are publicly available.
