# Supplementary material for: Tracking Population-Level Anxiety Using Search Engine Data: Ecological Study
Source: JMIR Form Res. 2023 Mar 22;7:e44055. doi: 10.2196/44055 (PMC10131769; doi:10.2196/44055)
Supplement: Multimedia Appendix 1 [file formative_v7i1e44055_app1.docx]

# Multimedia Appendix 1. Supplementary information.

**1. Survey fields:**

1. What are some things you might feel anxious about in the short-term?
2. What are some things you might feel anxious about in the long-term?
3. What are some things that your friends might feel anxious about in the short-term?
4. What are some things that your friends might feel anxious about in the long-term?
5. What is your current country of residence?

**2. Table A1. Anxiety themes included in the study, their time frame, and domains**

| Themes | Duration (long term vs. short term) | Domain (Personal\Family\Country) |
| --- | --- | --- |
| Accidents | Short | Family |
| Big test | Short | Personal |
| Breakup | Short | Personal |
| Cancer | Long | Family |
| Chronic illness | Long | Family |
| Cockroaches | Short | Personal |
| Covid19 | Long | Country |
| Diet | Long | Personal |
| Driving | Short | Personal |
| Drug addiction | Long | Family |
| Economic stability | Long | Family |
| Economy | Long | Country |
| Finances | Long | Family |
| Financial security | Long | Family |
| Financial stability | Long | Family |
| Food | Short | Family |
| Fiving birth | Short | Personal |
| Happiness | Long | Personal |
| Having kids | Long | Family |
| Insurance | Long | Family |
| Interviews | Short | Personal |
| Job | Long | Personal |
| Lizards | Short | Personal |
| Moving house | Short | Family |
| My health | Long | Personal |
| My reputation | Short | Personal |
| Political climate | Long | Country |
| Politics | Long | Country |
| Pregnancy | Short | Personal |
| Public image | Long | Personal |
| Rejection | Short | Personal |
| Retirement | Long | Family |
| Social interactions | Short | Personal |
| Taxes | Short | Family |
| Too much work | Short | Personal |
| Travel | Short | Family |
| Violence | Short | Country |

**3. Definitions for country-level attributes:**

1. *Freedom score*

The state of human freedom in a country based on a broad measure encompassing personal, civil and economic freedom.

1. *Religiosity*

Degree of religious involvement or commitment.

1. *Fragile state*

A state is characterized by weak state capacity or legitimacy, leaving citizens vulnerable to a range of shocks.

1. *Cultural dimensions*

Power distance: the degree to which the less powerful members of a society accept and expect that power is distributed unequally.

Uncertainty avoidance*:* the degree to which the members of a society feel uncomfortable with uncertainty and ambiguity.

Individualism/collectivism*:* individualism is defined as a preference for a loosely-knit social framework in which individuals are expected to take care of only themselves and their immediate families; collectivism represents a preference for a tightly-knit framework in society in which individuals can expect their relatives or members of a particular ingroup to look after them in exchange for unquestioning loyalty.

Masculinity/femininity*:* masculinity represents a preference in society for achievement, heroism, assertiveness, and material rewards for success; femininity stands for a preference for cooperation, modesty, caring for the weak and quality of life.

Long/short-term orientation*:* societies who score low on this dimension prefer to maintain time-honoured traditions and norms while viewing societal change with suspicion; those with a culture which scores high take a more pragmatic approach: they encourage thrift and efforts in modern education as a way to prepare for the future.

Indulgence/restraint*:* indulgence stands for a society that allows relatively free gratification of basic and natural human drives related to enjoying life and having fun; restraint stands for a society that suppresses gratification of needs and regulates it by means of strict social norms.

**4. Validating data and methods used**

**Figure A1** shows the fraction of times that anxiety themes identified by crowdsourced participants were mentioned in Bing queries in conjunction with words related to anxiety (e.g., “anxious about financial burden”), as a function of the fraction of times they were mentioned in Bing queries in any context (e.g., “financial burden.”). Spearman correlation between the two is 0.86 (p<10^-10^), implying a very strong correlation between the anxiety themes identified through crowdsourcing when queried on Bing with and without context. Data points closer to the fitted line are those themes which we assume are indicative of a query about anxiety even when they are queried without a specific mention of anxiety (e.g., giving birth).


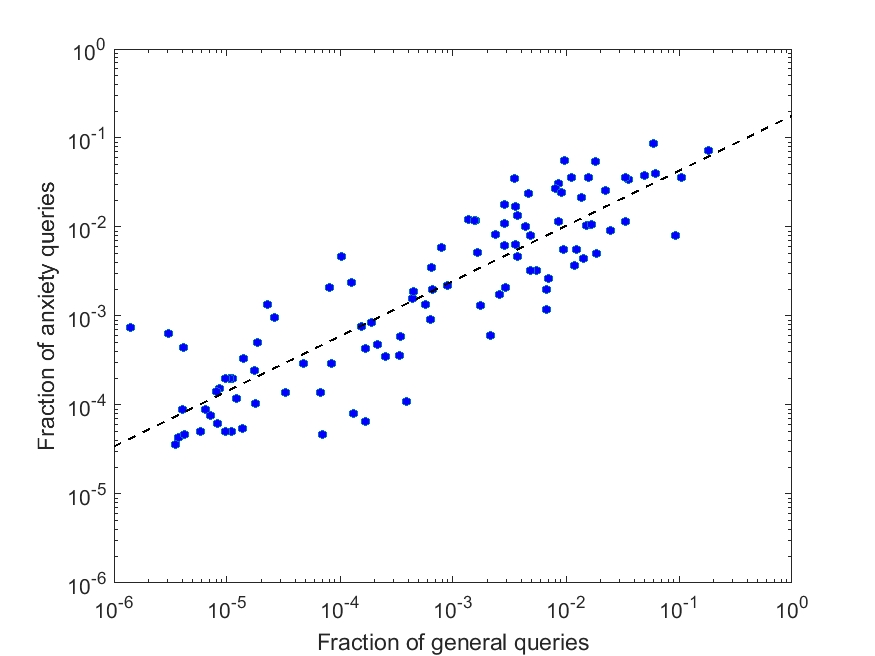


*Figure A1: Correlation between the fraction of mentions of anxiety themes/terms in general and anxiety-specific contexts. The dotted line is a linear fit to the data.*

1. **Correlation of volumes of anxiety themes across countries**

**Figure A2** shows that some geographically proximate countries are more similar on volume of query terms (e.g., Germany and Italy).


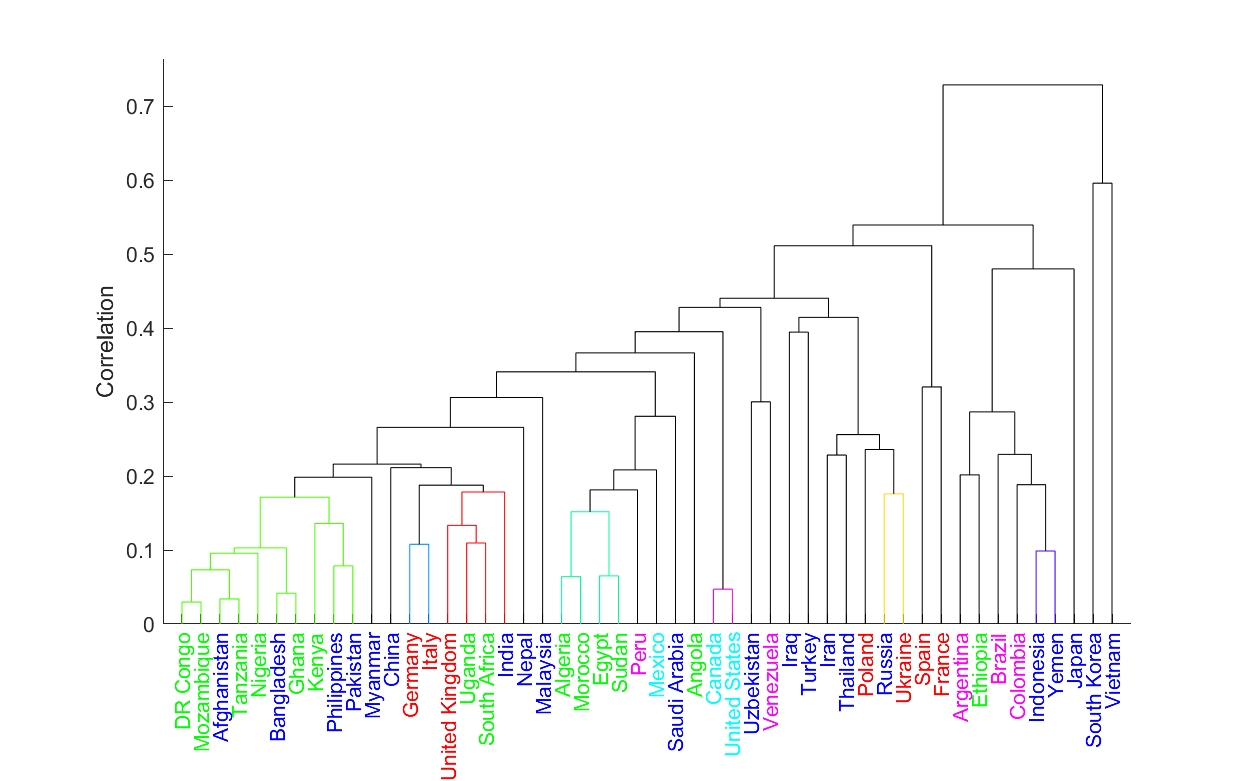


*Figure A2: Dendrogram of the similarity between countries on query volume for different anxiety themes. Two countries are more similar (and thus grouped lower in the graph) if query volume for different anxiety themes is similar among them. Countries are colour coded by continent.*

**Table A2 The most voluminous anxiety them by country and year**

| **Country** | **2004** | **2005** | **2006** | **2007** | **2008** | **2009** | **2010** | **2011** | **2012** | **2013** | **2014** | **2015** | **2016** | **2017** | **2018** | **2019** | **2020** | **Income Group** | **Region** |
| --- | --- | --- | --- | --- | --- | --- | --- | --- | --- | --- | --- | --- | --- | --- | --- | --- | --- | --- | --- |
| Afghanistan | job | job | job | job | job | job | job | job | job | job | job | job | job | job | job | job | covid19 | Low | South Asia |
| Algeria | having kids | having kids | having kids | having kids | having kids | having kids | having kids | having kids | having kids | having kids | having kids | having kids | having kids | having kids | having kids | having kids | having kids | Upper-middle | Middle East & North Africa |
| Angola | economy | politics | economy | having kids | economy | economy | pregnancy | pregnancy | pregnancy | pregnancy | pregnancy | pregnancy | pregnancy | pregnancy | pregnancy | pregnancy | covid19 | Upper-middle | Sub-Saharan Africa |
| Argentina | having kids | having kids | having kids | having kids | having kids | having kids | having kids | having kids | having kids | having kids | having kids | having kids | having kids | having kids | having kids | having kids | covid19 | High | Latin America & Caribbean |
| Bangladesh | job | job | job | job | job | job | job | job | job | job | job | job | job | job | job | job | covid19 | Lower-middle | South Asia |
| Brazil | my health | my health | my health | my health | my health | my health | my health | my health | my health | my health | my health | cockroaches | cockroaches | cockroaches | cockroaches | cockroaches | covid19 | Upper-middle | Latin America & Caribbean |
| Canada | my health | my health | my health | my health | my health | my health | job | job | job | job | job | job | job | job | having kids | job | covid19 | High | North America |
| China | insurance | insurance | insurance | insurance | insurance | insurance | insurance | pregnancy | pregnancy | pregnancy | having kids | having kids | having kids | having kids | having kids | having kids | covid19 | Upper-middle | East Asia & Pacific |
| Colombia | my health | having kids | having kids | having kids | having kids | having kids | having kids | having kids | having kids | having kids | having kids | having kids | having kids | having kids | having kids | having kids | having kids | Upper-middle | Latin America & Caribbean |
| DR Congo | having kids | economy | politics | having kids | politics | having kids | politics | politics | politics | having kids | politics | politics | politics | politics | politics | politics | covid19 | Low | Sub-Saharan Africa |
| Egypt | having kids | having kids | having kids | having kids | having kids | having kids | having kids | having kids | having kids | having kids | having kids | having kids | having kids | having kids | having kids | having kids | having kids | Lower-middle | Middle East & North Africa |
| Ethiopia | having kids | having kids | having kids | having kids | having kids | having kids | having kids | having kids | having kids | having kids | having kids | having kids | having kids | having kids | having kids | having kids | covid19 | Low | Sub-Saharan Africa |
| France | having kids | having kids | having kids | having kids | travel | travel | travel | travel | travel | having kids | having kids | having kids | having kids | having kids | having kids | having kids | covid19 | High | Europe & Central Asia |
| Germany | travel | job | job | job | having kids | having kids | having kids | job | job | job | job | job | having kids | having kids | having kids | having kids | having kids | High | Europe & Central Asia |
| Ghana | job | job | job | job | job | job | job | job | job | job | job | job | job | job | pregnancy | job | covid19 | Lower-middle | Sub-Saharan Africa |
| India | job | job | job | job | job | job | job | job | job | job | job | job | job | job | job | job | covid19 | Lower-middle | South Asia |
| Indonesia | my health | my health | my health | my health | my health | my health | my health | my health | my health | my health | my health | my health | my health | my health | my health | my health | my health | Lower-middle | East Asia & Pacific |
| Iran | having kids | having kids | having kids | having kids | having kids | having kids | having kids | having kids | having kids | having kids | having kids | having kids | having kids | having kids | having kids | having kids | having kids | Upper-middle | Middle East & North Africa |
| Iraq | taxes | travel | taxes | travel | travel | travel | travel | pregnancy | pregnancy | pregnancy | pregnancy | pregnancy | pregnancy | pregnancy | pregnancy | pregnancy | pregnancy | Upper-middle | Middle East & North Africa |
| Italy | travel | travel | travel | travel | travel | travel | travel | pregnancy | pregnancy | pregnancy | pregnancy | pregnancy | pregnancy | having kids | having kids | having kids | covid19 | High | Europe & Central Asia |
| Japan | insurance | insurance | insurance | insurance | insurance | finances | finances | insurance | insurance | insurance | insurance | insurance | insurance | having kids | having kids | having kids | having kids | High | East Asia & Pacific |
| Kenya | job | job | job | job | job | job | job | job | job | job | job | job | job | job | job | job | covid19 | Lower-middle | Sub-Saharan Africa |
| Malaysia | job | my health | my health | my health | my health | job | job | job | having kids | having kids | having kids | having kids | having kids | having kids | having kids | having kids | covid19 | Upper-middle | East Asia & Pacific |
| Mexico | having kids | having kids | having kids | having kids | having kids | having kids | having kids | having kids | having kids | having kids | having kids | having kids | having kids | having kids | having kids | having kids | having kids | Upper-middle | Latin America & Caribbean |
| Morocco | having kids | having kids | having kids | having kids | having kids | having kids | having kids | having kids | having kids | having kids | having kids | pregnancy | pregnancy | pregnancy | pregnancy | having kids | covid19 | Lower-middle | Middle East & North Africa |
| Mozambique | having kids | economy | economy | economy | economy | politics | pregnancy | pregnancy | pregnancy | pregnancy | pregnancy | pregnancy | pregnancy | pregnancy | pregnancy | pregnancy | covid19 | Low | Sub-Saharan Africa |
| Myanmar | job | job | job | job | job | job | job | job | job | job | job | job | job | job | job | job | covid19 | Lower-middle | East Asia & Pacific |
| Nepal | job | job | job | job | job | job | job | job | job | job | job | job | job | job | job | job | covid19 | Low | South Asia |
| Nigeria | job | job | job | job | job | job | job | job | job | job | job | job | job | job | pregnancy | pregnancy | pregnancy | Lower-middle | Sub-Saharan Africa |
| Pakistan | having kids | job | job | job | job | job | job | job | job | having kids | job | having kids | having kids | job | job | job | having kids | Lower-middle | South Asia |
| Peru | having kids | having kids | having kids | having kids | having kids | having kids | having kids | having kids | having kids | having kids | having kids | having kids | having kids | having kids | having kids | having kids | having kids | Upper-middle | Latin America & Caribbean |
| Philippines | job | job | job | job | job | job | job | job | job | job | job | job | job | job | having kids | having kids | covid19 | Lower-middle | East Asia & Pacific |
| Poland | taxes | taxes | taxes | taxes | taxes | taxes | taxes | taxes | taxes | taxes | taxes | taxes | taxes | taxes | taxes | taxes | taxes | High | Europe & Central Asia |
| Russia | job | job | job | job | job | job | job | job | job | job | job | job | job | having kids | having kids | having kids | having kids | High | Europe & Central Asia |
| Saudi Arabia | having kids | having kids | having kids | having kids | having kids | having kids | having kids | having kids | having kids | having kids | having kids | having kids | having kids | having kids | having kids | having kids | having kids | HIgh | Middle East & North Africa |
| South Africa | job | job | job | job | job | job | job | job | job | job | job | having kids | having kids | having kids | having kids | having kids | covid19 | Upper-middle | Sub-Saharan Africa |
| South Korea | my health | cancer | cancer | cancer | cancer | cancer | cancer | cancer | cancer | cancer | cancer | cancer | cancer | finances | having kids | finances | covid19 | High | East Asia & Pacific |
| Spain | travel | travel | travel | travel | having kids | having kids | having kids | having kids | having kids | having kids | having kids | having kids | having kids | having kids | having kids | having kids | covid19 | High | Europe & Central Asia |
| Sudan | my health | my health | my health | having kids | having kids | having kids | having kids | having kids | having kids | having kids | having kids | having kids | having kids | pregnancy | pregnancy | pregnancy | pregnancy | Lower-middle | Sub-Saharan Africa |
| Tanzania | job | job | job | job | job | job | job | job | job | job | job | having kids | having kids | having kids | having kids | having kids | covid19 | Low | Sub-Saharan Africa |
| Thailand | food | food | food | food | food | food | food | food | food | food | food | food | food | food | food | food | food | Upper-middle | East Asia & Pacific |
| Turkey | taxes | taxes | having kids | having kids | having kids | having kids | having kids | having kids | having kids | having kids | having kids | having kids | having kids | having kids | having kids | having kids | having kids | Upper-middle | Europe & Central Asia |
| Uganda | job | job | job | job | job | job | job | job | job | job | job | job | job | job | job | job | covid19 | Low | Sub-Saharan Africa |
| Ukraine | economy | economy | economy | economy | finances | finances | finances | finances | finances | finances | finances | finances | finances | finances | finances | finances | finances | Lower-middle | Europe & Central Asia |
| United Kingdom | job | job | job | job | job | job | job | job | job | job | job | job | job | job | job | job | covid19 | High | Europe & Central Asia |
| United States | job | having kids | having kids | job | job | job | job | job | job | having kids | job | job | having kids | having kids | having kids | having kids | covid19 | High | North America |
| Uzbekistan | job | having kids | job | job | having kids | having kids | having kids | having kids | having kids | having kids | having kids | having kids | having kids | having kids | having kids | having kids | having kids | Lower-middle | Europe & Central Asia |
| Venezuela | having kids | having kids | having kids | having kids | having kids | having kids | having kids | having kids | having kids | having kids | having kids | having kids | having kids | having kids | having kids | having kids | having kids | High | Latin America & Caribbean |
| Vietnam | travel | travel | travel | insurance | insurance | insurance | insurance | insurance | insurance | insurance | interviews | insurance | insurance | insurance | insurance | insurance | insurance | Lower-middle | East Asia & Pacific |
| Yemen | having kids | having kids | having kids | having kids | having kids | having kids | having kids | having kids | having kids | having kids | having kids | having kids | having kids | having kids | having kids | having kids | having kids | Lower-middle | Middle East & North Africa |
